# Deep Learning Deepens the Analysis of Alternative Splicing

**DOI:** 10.1016/j.gpb.2019.05.001

**Published:** 2019-05-14

**Authors:** Xudong Zou, Xin Gao, Wei Chen

**Affiliations:** 1 Department of Biology, Southern University of Science and Technology, Shenzhen 518055, China; 2 Computational Bioscience Research Center (CBRC), Computer Electrical and Mathematical Sciences and Engineering (CEMSE) Division, King Abdullah University of Science and Technology (KAUST), Thuwal 23955, Saudi Arabia

The ever-increasing high-volume and high-dimensional genomics data on the one hand challenge traditional data analysis approaches, and on the other hand provide ample opportunities for developing novel analytic strategies. In recent years, deep learning has been driving the next wave of artificial intelligence and machine learning. Now, Yi Xing’s lab reported DARTS [1], a novel computational framework that leverages the power of both deep learning and Bayes hierarchical framework for differential alternative splicing (AS) analysis. Trained on the huge volume of publicly-available RNA-seq datasets, DARTS could largely increase the accuracy of AS analysis, in particular for those with low sequencing depth, by taking both genomic features and expression levels of RNA-binding proteins (RBPs) into consideration.

## RNA-seq based AS analysis

In higher eukaryotes, the vast majority of protein-coding genes are transcribed into precursor mRNA (pre-mRNA) containing exons and introns that need to be removed by the splicing machinery to generate mature mRNA. Often the transcript can be spliced in different ways, leading to a different combination of exons. AS contributes to the variety of the cellular proteome as well as to the fine tuning of gene expression levels at the post-transcriptional level. The regulation of AS is mediated by the interaction between *cis*-elements around the splicing site in both exonic and intronic regions, and *trans*-acting factors that bind to specific *cis*-elements. It has been shown that AS plays critical roles in a variety of physio-pathological processes.

In the last decade, the amount of RNA-seq data has soared, which provides valuable resources for extensive studies of transcriptional and post-transcriptional regulation. In addition to providing the information on RNA abundance, RNA-seq data could also be used to infer the AS pattern, and more often to identify the differential AS between different samples, such as those from different developmental stages, normal *vs*. disease, as well as control *vs*. treatment. For the latter, many computational methods have been developed. The common strategy underlying these methods is to use the number of RNA-seq reads exclusively supporting either isoform to estimate an abundance ratio between the two spliced isoforms, *i.e.*, inclusion and exclusion isoform, and then perform a statistical test to determine whether the splicing pattern between the two samples is significantly different or not. Although implemented with different statistical frameworks, all these methods would encounter high uncertainty for the splicing events sampled with low sequencing coverage. Therefore, the sensitivity is rather limited in detecting differential AS for the lowly-expressed genes. Moreover, many currently available RNA-seq datasets originally designed only for differential analysis of gene expression are often of low sequencing depth, which is insufficient for AS analysis even for moderately-expressed genes. Harnessing these valuable resources for AS studies warrants novel analytic strategies.

## Deep-learning and its application in AS analysis

Deep learning has recently reemerged in various fields (*e.g.*, image recognition and language processing) with great success. Unlike the traditional machine learning algorithms, deep learning trains both the feature extractor and the classifier simultaneously. The high model complexity caused by the extraordinarily large number of parameters makes deep learning models data-hungry. Such high model flexibility, on the other hand, together with the powerful optimization algorithms, enables deep learning to achieve the state-of-the-art performance on a wide spectrum of applications where large datasets are available, such as computer vision, natural language processing, and genomics.

The seminal work on developing deep learning methods to decipher the splicing code was done by Leung and colleagues [2]. They studied the tissue-specific splicing code of five tissues in mice. For each exon, their model takes 1393 manually-extracted features, including those from exon, neighboring intron, adjacent exon, as well as tissue type indicators, as inputs, and predicts the range (low, medium, or high) of the percentage of isoform including that exon (the Percent Splicing In (PSI) value) and ΔPSI between two tissues. In a follow-up study, Xiong et al. improved the model to predict the exact value of PSI by using the same set of features and applied the model to detect splicing-affecting variants that are associated with human diseases [3]. Recently, Bretschneider et al. further developed four different deep learning models to predict alternative acceptor sites and alternative donor sites [4]. In contrast to the previous work from the same lab, Bretschneider et al. leveraged the power of deep learning to automatically extract important features for raw DNA sequences and built models to simultaneously predict the PSI values of all the alternative sites with an accuracy of 70%. More recently, Jaganathan et al. also developed a deep learning method to predict whether each position in the transcript could function as a splice donor or a splice acceptor, or neither of them [5]. Compared to previous methods that relied on human-designed features, or have only considered short nucleotide windows adjoining exon–intron boundaries, this method learns splicing determinants from 10,000 nucleotides around each candidate position, with a 95% top-k accuracy. Nonetheless, AS is regulated by the interplay between *cis*-regulatory elements and *trans*-acting factors, these deep learning models were mostly focused only on the contribution of *cis-*sequence features and have largely ignored *trans*-environment. As a result, they could not, for instance, tell any differential AS between two samples with the same genomic sequences but under different conditions.

## DARTS—deep-learning augmented RNA-seq analysis of transcript splicing

The new tool, DARTS, mainly consists of two components, *i.e.*, a deep learning model (DARTS DNN) to estimate the prior probability and a likelihood estimator (DARTS BHT) based on the prior probability as well as RNA-seq read counts. Before training DARTS DNN, large-scale RNA-seq data are analyzed first by DARTS BHT with uninformative prior to generate a high-confidence labeled training dataset that contains both differential splicing and unchanged splicing between conditions. Then the labeled training dataset is used for training DARTS DNN. In contrast to the aforementioned deep learning-based AS methods, this deep learning module incorporates not only the *cis*-elements from the primary genomic sequences but also the *trans*-elements represented by the expression level of 1498 splicing-relevant RBPs. Zhang et al. first evaluated the performance of DARTS on the test data corresponding to leave-out RBPs and showed that DARTS outperformed the baseline methods. They then applied DARTS on two cell lines to infer cell-type-specific splicing events, in which they found that the performance of DARTS BHT with an informative prior probability is better than that without the prior, demonstrating that incorporating DNN prediction as an informative prior improves the performance of DARTS BHT in detecting differential splicing. To further demonstrate the power of DARTS DNN on other RNA-seq datasets, they trained three DARTS DNN models using ENCODE data only, Roadmap data only, and their combination, respectively. They found that the model trained on ENCODE data has high predictive power for the ENCODE leave-out datasets, but modest predictive power for Roadmap leave-out datasets, and *vice versa*, while the model trained on the combination of both datasets has the best performance. Furthermore, they extended DARTS DNN to other types of AS events, *i.e.*, alternative 5′ or 3′ splice sites and retained introns, and they also achieved a high prediction accuracy. Finally, they applied DARTS to investigate the change of AS pattern during the epithelial-mesenchymal transition (EMT) using the previously published RNA-seq dataset [6]. Using DARTS, they were not only able to predict high-confidence differential versus unchanged splicing events during the EMT, but also uncover differential AS events from lowly-expressed genes. Importantly, the latter could successfully be experimentally validated, again demonstrating the improved accuracy of DARTS on AS with lower sampling depth.

The major innovation of DARTS lies in two aspects. (1) DARTS combines a deep learning model with Bayes hierarchical framework: the former provides the latter *a prior* based on learned knowledge about each AS event in a specific sample, while the latter further integrates the information from RNA-seq data. (2) The deep learning model within DARTS framework for the first time takes both *cis*-elements and *trans*-factors into consideration, which improves differential AS detection between conditions.

## Discussion

There are yet some directions for further development of DARTS. First, although DARTS can theoretically capture the *cis*–*trans* interactions, such association requires a prohibitively large number of input combinations. Second, DARTS is trained on invariant genomic sequences from different samples, and thus could not capture the splicing landscape of sequence variants. Third, the performance of DARTS may be further improved by incorporating increased lengths of flanking regions or more *cis*-features. However, it requires more data and sophisticated feature engineering to obtain a better model.

Other than AS, alternative polyadenylation (APA) is also a key, but less-well studied step in RNA processing. And conceptually, similar to AS, the regulation of APA is also mediated by *cis*–*trans* interaction. Therefore, APA regulation could be treated as a similar problem and accordingly investigated with a similar strategy. Xia et al. recently developed a robust, poly(A) signal (PAS) motif-agnostic, and transferable deep learning model to differentiate true PASs from false ones [7]. The ideas of DARTS could potentially be applied to combine the power of novel deep learning based computational algorithms and RNA-seq based experimental data for APA analysis.

## Competing interests

The authors have declared no competing interests.
